# Cost and Cost-Effectiveness of mHealth Interventions to Promote Uptake of Antenatal Care (ANC) in Low- and Middle-Income Countries: A Scoping Review

**DOI:** 10.7759/cureus.110807

**Published:** 2026-06-14

**Authors:** Elly Atuhumuza, Esther C Atukunda, Wilson Tumuhimbise, Mugyenyi R Godfrey, Angella Musiimenta, Vincent Batwala

**Affiliations:** 1 Community Health, Mbarara University of Science and Technology, Mbarara, UGA; 2 Research Department, StrongMinds, Kampala, UGA; 3 Pharmacy, Mbarara University of Science and Technology, Mbarara, UGA; 4 Information Technology, Mbarara University of Science and Technology, Mbarara, UGA; 5 Obstetrics and Gynaecology, Mbarara University of Science and Technology, Mbarara, UGA; 6 Research and Innovations, Angels Compassion, Mbarara, UGA; 7 Research and Graduate Training, Mbarara University of Science and Technology, Mbarara, UGA

**Keywords:** antenatal care, cost effectiveness analysis, low and middle country (lmic), maternal health, mobile health (mhealth)

## Abstract

Mobile health (mHealth) innovations are increasingly being considered to improve antenatal care (ANC) uptake in low- and middle-income countries (LMICs). However, there is limited evidence of their value for money, which limits policymakers' ability to make decisions about sustainability and scale. This scoping review synthesizes available evidence on the cost and cost-effectiveness of mHealth interventions aimed at promoting ANC uptake in LMICs. Following the Arksey and O’Malley framework and Preferred Reporting Items for Systematic Reviews and Meta-Analyses extension for Scoping Reviews (PRISMA-ScR) guidelines, we searched PubMed, Science Direct, and Google Scholar databases for peer-reviewed studies reporting economic evaluations. These included studies reporting cost-effectiveness, cost-benefit, or costing analyses of mHealth interventions targeting ANC, published between 2010 and 2025.

Screening and data extraction were performed by two independent reviewers. Data was charted and synthesized narratively, and findings were presented in tables. From the 1,249 records screened, 10 studies met the inclusion criteria: seven cost-effectiveness analyses and three costing studies. Studies were from South Asia and sub-Saharan Africa, including India (n=3), Bangladesh (n=2), and one each from Nigeria, South Africa, Ghana, Iraq, and Malawi. Interventions included short message service (SMS) reminders, mobile job aids for community health workers, and integrated digital platforms. Improvements were reported in ANC outcomes such as early registration, four or more visits, and iron-folic acid consumption. Costs ranged from $0.0225 per SMS in Iraq to $29.33 per user in Malawi. Incremental cost-effectiveness ratios varied from $21 to $568 per disability-adjusted life year averted and were below prevailing country-specific gross domestic product (GDP) per capita thresholds, though direct cross-study comparison is limited by differences in analytical perspective, time horizon, and modelling assumptions. Four studies adopted a societal perspective, four a program perspective, and one a health system perspective; five of the seven cost-effectiveness analyses used the Lives Saved Tool for modelling.

Evidence gaps identified included a lack of equity analyses, limited long-term sustainability data, and a need for standardized methodological approaches. Reviewed evidence suggests that mHealth interventions for ANC promotion in LMICs are a cost-effective approach to improving ANC uptake, although evidence is largely modelled rather than empirically observed and hence calls for cautious interpretation. The common use of the Lives Saved Tool across most cost-effectiveness studies suggests an emerging standardization in how mHealth cost-effectiveness is assessed. Some gaps remain, including the absence of equity analyses, limited long-term sustainability evidence, and underrepresentation of sub-Saharan Africa. Standardized cost-effectiveness analyses with a focus on equity are needed to ensure future mHealth investments align with universal health coverage goals and benefit women across all socioeconomic groups.

## Introduction and background

Timely antenatal care (ANC) is known to be associated with a reduction in adverse pregnancy outcomes, such as maternal and neonatal mortality, stillbirths, and low birth weight [1-3], yet coverage remains low in many low- and middle-income countries (LMICs). Whereas the World Health Organization (WHO) recommends at least eight ANC contacts to enable early detection and management of complications such as pre-eclampsia, anemia, and infections [4], ANC coverage is still reported against the older four-visit benchmark, and by this measure, only 50-65% of women complete four ANC visits, compared to over 96% in high-income countries [5]. Barriers such as financial constraints, long distances to health facilities, cultural norms, and a lack of awareness regarding the importance of early and consistent care contribute to this disparity [6], resulting in high maternal mortality, with over 260,000 women dying annually from pregnancy-related causes, the majority of whom are in LMICs [7]. To address these barriers, mHealth interventions have been used to improve ANC uptake and reduce disparities in care-seeking behavior [8]. Leveraging mobile phone coverage, even in remote areas, mHealth can potentially deliver low-cost health information, send appointment reminders, and provide tailored support directly to pregnant women and their families [8,9].

Whereas evidence from multiple studies in LMICs (including Bangladesh, Malawi, and South Africa) shows that mHealth programs can improve ANC attendance by 20-30% and encourage earlier care seeking [10-12], translating these findings into scalable programs requires reliable economic evidence. There is a scarcity of rigorous cost-effectiveness analysis studies, yet this evidence is essential for policymakers to make better investment decisions between mHealth and conventional service delivery models [13]. Also, many studies are short-term pilots, and this leaves the question of long-term sustainability and true scale-up costs unanswered [14]. Furthermore, the cost-effectiveness of mHealth interventions is context dependent and influenced by local factors such as mobile network coverage, user engagement, and health system readiness [15,16]. The lack of clear evidence makes it harder to integrate mHealth into national maternal health strategies. This evidence gap must be addressed because improving maternal health is necessary for achieving Sustainable Development Goal 3 as well as Universal Health Coverage commitments [17]. Furthermore, as donor funding for global health becomes increasingly constrained [18], robust evidence is important to determine whether these investments will be sustained or abandoned.

Assessing cost-effectiveness requires a threshold against which value for money can be benchmarked. The World Health Organization Choosing Interventions that are Cost-Effective (WHO-CHOICE) framework, which uses one-time gross domestic product (GDP) per capita per disability-adjusted life year (DALY) averted as the threshold for a highly cost-effective intervention, has been widely applied in LMIC settings [15]. However, this threshold has been questioned, with some economists arguing that opportunity-cost-based thresholds, reflecting what a health system must forgo to fund a new intervention, are more appropriate in resource-constrained settings [15,19]. Understanding which threshold is applied is therefore important in interpreting and comparing cost-effectiveness findings across studies.

This scoping review aims to synthesize evidence on the costs and cost-effectiveness of mHealth-enabled interventions for promoting uptake of ANC in LMICs, focusing on examining intervention types, cost components, effectiveness outcomes, and economic evaluation methods, as well as identifying gaps to inform future intervention design and evaluations.

## Review

Methodology

This scoping review was conducted following the scoping studies framework developed by Arksey et al. [20] and later revised by Levac et al. [21]. All recommended steps were followed except for stakeholder consultation. Due to the exploratory nature of the review, critical appraisal of individual studies (e.g., using quality assessment checklists) was not performed. The focus was on mapping the range of evidence, including variations in study design and methodology, rather than formally assessing the risk of bias or methodological quality of each included study. The reporting adhered to the Preferred Reporting Items for Systematic Reviews and Meta-Analyses extension for Scoping Reviews (PRISMA-ScR) guidelines [22]. No review protocol was registered before conducting this scoping review.

Stage One: Identification of the Research Question

The research team formulated the primary research question as: “What evidence exists regarding the cost and cost-effectiveness of mHealth interventions designed to improve ANC uptake in developing countries?” This question was broken down into the following secondary questions: (1) the types of mHealth strategies [e.g., short message service (SMS), mobile apps, digital platforms] that have been economically evaluated; (2) the cost components and analytical perspectives used; (3) the ANC-related effectiveness outcomes used to measure intervention impact; (4) the methodological approaches used in the economic evaluations, including study design and modelling techniques; and (5) the key gaps in the existing body of evidence included: the types of economic analyses conducted, the range of costs considered, and the methodologies used across different studies. This approach aligns with the main purpose of scoping reviews, which is to examine the extent, range, and nature of research activity, as well as identify research gaps in the existing literature [21].

Stage Two: Identification of Relevant Studies

**Figure 1:**
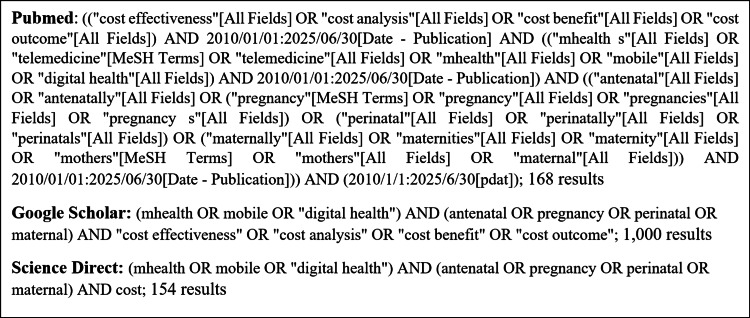
Search strategy for identifying relevant studies

We searched three databases. These include PubMed, Science Direct, and Google Scholar. The search was restricted to published peer-reviewed literature in line with the inclusion criteria; therefore, grey literature sources were not included in this review. The search string for PubMed included a publication date filter, while Science Direct and Google Scholar searches were filtered separately for studies published between January 2010 and June 2025. The search strategy combined terms across three themes: (1) mHealth/digital health technologies (e.g., mHealth, telemedicine, mobile health), (2) maternal/pregnancy-related care (e.g., antenatal, pregnancy, maternal), and (3) economic evaluation (e.g., cost-effectiveness, cost analysis, cost benefit). The full search strings for each database are provided in Figure 1.

Stage Three: Study Selection

In selecting studies, we used a two-phase screening approach, with two independent reviewers. In the first phase, all identified records were imported into Zotero 7 for Windows (Corporation for Digital Scholarship, Falls Church, USA). Here, duplicates were removed and records screened based on title and abstract. mHealth was defined as “medical and public health practice supported by mobile devices, such as mobile phones, patient monitoring devices, personal digital assistants (PDAs), and other wireless devices” [23]. Both demand (client-facing) and supply (provider-facing) mHealth interventions were considered. Studies were included if they: (1) focused on pregnant women in LMICs, encompassing low, lower-middle, and upper-middle income countries, as defined by the World Bank [24], (2) evaluated an mHealth intervention specifically targeting antenatal care uptake or utilization, or (3) reported cost or cost-effectiveness analysis. Studies were excluded if they: (1) lacked any economic evaluation component, (2) were conducted in high-income country settings, (3) were not published in English, or (4) were systematic reviews, meta-analyses, or other secondary literature syntheses.

In the second phase, full-text articles from all studies that were potentially eligible were retrieved and assessed in more detail using the same inclusion/exclusion criteria. At both screening phases, any disagreements between reviewers were resolved through discussion. Where necessary, a third senior reviewer was consulted. The study selection process was documented using a PRISMA flow diagram. The PRISMA flow diagram presents the number of records identified, screened, excluded, and the final number included in the review, along with the reasons for exclusion at the full-text stage [22].

Stage Four and Five: Data Charting, Collating, Summarizing, and Reporting Results

Data from the included studies were charted using a Microsoft Excel-based data extraction form that was piloted on a sample of articles. The extraction form included the following fields: author, year, and country; study design and sample size; mHealth intervention type, description, and comparator; analytical perspective, time horizon, and discount rate; cost components and cost estimates; effectiveness outcomes and incremental cost-effectiveness ratios (ICERs); and conclusions relevant to policy. The findings were summarized narratively. Tables were used to present an overview of the evidence and to map the identified gaps, types of interventions evaluated, and methodological limitations in the existing economic evaluations.

Results

Literature Search

**Figure 2:**
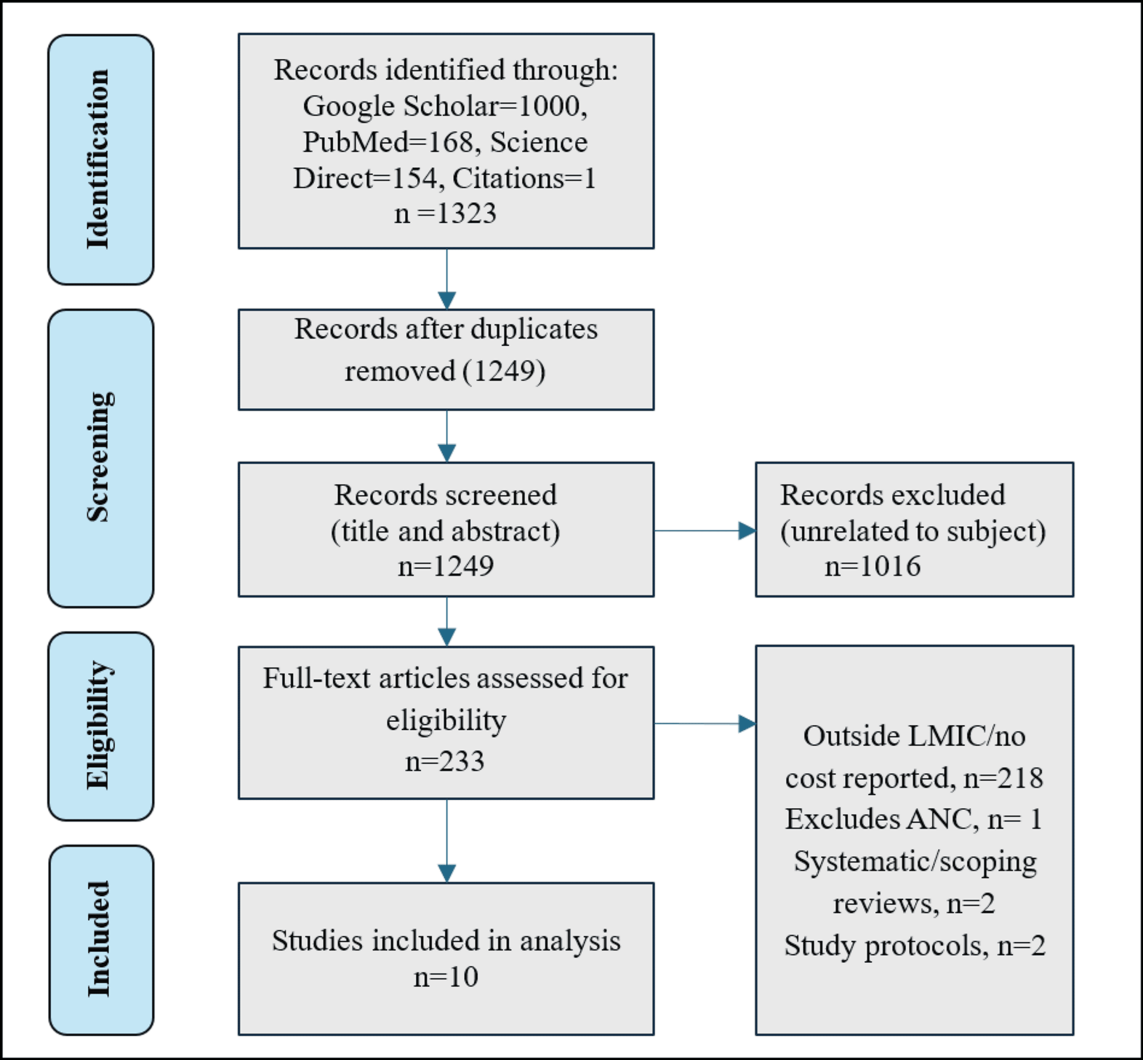
Flow diagram of selected studies LMIC: low- and middle-income countries; ANC: antenatal care.

A total of 1,323 records were identified from three databases and citation searching: Google Scholar (n=1,000), PubMed (n=168), and Science Direct (n=154). One additional record was identified through citation searching. After removing 74 duplicates, 1,249 records were screened by title and abstract. Out of 1,249, 1,016 records were excluded because they did not meet any of the criteria. Full-text screening was performed on 233 articles, of which 223 were excluded for reasons including being conducted outside LMICs or having no costs reported (218), being systematic or scoping reviews (2), not focusing on ANC (1), or being study protocols (2). Ten studies were finally included for analysis. The PRISMA flow diagram detailing the study selection process is presented in Figure 2. 

General Characteristics

The included studies evaluated a range of mHealth interventions designed to improve various maternal and child health (MCH) outcomes that included antenatal care (ANC). These studies were spread across South Asia and sub-Saharan Africa. Ten studies from seven countries were selected, including India (n=3), Bangladesh (n=2), Nigeria (n=1), South Africa (n=1), Ghana (n=1), Iraq (n=1), and Malawi (n=1). Of these, seven were cost-effectiveness analyses [10,12,13,16,25-27], and three were costing studies [11,28,29]. Detailed characteristics of the studies are presented in Table 1.

**Table 1:** General characteristics of included studies LMIC: low- and middle-income countries; CHW: community health worker; TeCHO+: Technology for Community Health Operations Plus; HR: ; ANC: antenatal care; DALY: disability-adjusted life year; eMamta: Electronic Mother and Child Tracking System; CommCare™: mobile job aid platform; SMS: short message service; mCARE: mobile-based pregnancy surveillance and reminder system; ASHAs: Accredited Social Health Activists; MNCH: maternal, newborn, and child health; ReMiND: Reducing Maternal and Newborn Deaths; CCPF: Chipatala cha pa Foni ("Health Center by Phone"); CEA: cost-effectiveness analysis; MCH: maternal and child health; PNC: postnatal care; RCT: randomized controlled trial; LiST: Lives Saved Tool; UP: Uttar Pradesh.

Study	Country, LMIC classification, setting, sample size & study design	Intervention type	Intervention description & comparator	Cost components	Effectiveness outcomes
Cost-effectiveness analyses (n=7)					
Saha et al. [26]	India; Lower-middle income; Community-based (Gujarat); unspecified sample size; Pre-post with decision-tree modeling	CHW mobile/web app	Intervention: TeCHO+ mobile/web app for ASHAs: real-time data entry, high-risk case alerts, supervision dashboards. Comparator: eMamta (prior paper-based/partial digital system)	Start-up (phones, training); Implementation (monitoring, human resource, helpline); Service delivery (ANC, immunization, delivery)	DALYs averted; ANC coverage; High-risk case detection
Bowser et al. [13]	Nigeria; Lower-middle income; Facility-based (Abuja, Nassarawa); 20 facilities (10 intervention, 10 control); Matched case-control	Mobile job aid for health workers	Intervention: CommCare™ mobile job aid: task management, decision support, service tracking. Comparator: Matched facilities without mHealth job aid	Program costs (personnel, training, devices, software); Service delivery (ANC drugs, lab tests, facility births); Societal costs (transport, lost wages)	Lives saved; DALYs averted; ANC coverage; Facility delivery rates
Prinja et al. [16]	India; Lower-middle income; Community-based (Kaushambi district, UP); Household surveys (unspecified sample size); Quasi-experimental with decision-tree modeling (10 years)	CHW mobile app	Intervention: ReMiND mobile app for ASHAs: pregnancy tracking, ANC guidance, complication identification, referral facilitation. Comparator: Standard government MCH program without mHealth	Startup (software, training, phones); Implementation (monitoring, services); Societal costs (household expenditures)	Maternal deaths averted; Neonatal deaths averted; DALYs averted; ANC coverage; Complication identification
LeFevre et al. [12]	South Africa; Upper-middle income; Facility-based (Johannesburg); 177 women; Retrospective case-control with modeling (5 years)	SMS messaging to pregnant women	Intervention: Bi-weekly SMS with gestational age-specific health information (danger signs, nutrition, ANC reminders). Comparator: No messaging service	Program costs (software, SMS, personnel); Health system costs (provider time for registration, ANC/PNC); User costs (transport, lost wages, etc.)	Proportion of women attending ≥4 ANC visits; Childhood immunization completion; lives saved; DALYs averted
Willcox et al. [27]	Ghana; Lower-middle income; Community + facility hybrid (Goma West); Single district; RCT	Dual-component: voice messages (demand) + client data app (supply)	Intervention: (1) Mobile Midwife: automated voice messages; (2) Client Data Application: digitized records, care alerts. Comparator: Paper-based status quo	Development (design, technology); Start-up (training, equipment, mobilization); Implementation (personnel, monitoring, phone replacements, airtime)	Maternal lives saved; Child lives saved; Stillbirths averted; DALYs averted; Skilled birth attendance; Facility delivery; Measles immunization; ANC attendance
Jo et al. [25]	Bangladesh; Lower-middle income; Community-based (rural, modeled scale-up to 10M population); Model-based; Model-based CEA (LiST)	Digital health intervention package (surveillance + reminders)	Intervention: mCARE package: mobile-based pregnancy tracking for CHWs, automated SMS reminders for ANC/delivery/PNC, CHW home-visit reminders. Comparator: Paper-based status quo	Training, supervision, technology, mobile phone services, and CHW time	ANC coverage; Maternal deaths averted; Neonatal deaths averted; Stillbirths averted; DALYs averted
Jo et al. [10]	Bangladesh; Lower-middle income; Community-based (Gaibandha District); 610 pregnant women (330 int, 280 control); Quasi-experimental	Digital health intervention (two intensities)	Intervention (comprehensive): mCARE with digital surveillance + SMS reminders + CHW home-visit reminders. Comparator (basic): mCARE with digital surveillance only	Development (system, phones); Start-up (training, outreach); Implementation (SMS, home-visits, supervision)	Neonatal deaths averted; DALYs averted; ANC use; Facility delivery
Cost analyses (n=3)					
Alhaidari et al. [28]	Iraq; Upper-middle income; Facility-based (Baghdad, ANC clinics); 250 women (100 int, 150 control); Non-randomised controlled	SMS reminders + health advice	Intervention: Weekly SMS: ANC visit reminders, health advice, nutrition/lifestyle education; Toll-free helpline. Comparator: Standard care (no SMS)	Direct: SMS messaging ($0.0225/message, $159.89 total); Indirect: Provider time (not monetized)	Median ANC visits; Program acceptability
Prinja et al. [29]	India; Lower-middle income; Community-based (two blocks, UP); Two blocks (unspecified sample size); Economic costing analysis	CHW mobile app (costing study)	Intervention: ReMiND mobile app for ASHAs: pregnancy tracking, counseling, MNCH service delivery support. Comparator: None	Program implementation (software, training, equipment, mobile data, staff); Health system (supervision, service delivery)	None
Larsen-Cooper et al. [11]	Malawi; Lower-middle income; Community-based (Balaka District); 9,798 users; Cost-outcome analysis	Toll-free hotline + voice/SMS tips	Intervention: Chipatala cha pa Foni (CCPF): toll-free maternal health hotline + voice/SMS tips and reminders. Comparator: None	Total program costs (allocated 50% to maternal health)	None

Interventions Types

The included studies evaluated a range of mHealth strategies, with many incorporating multiple delivery channels. The interventions focused on improving supply-side capabilities of health workers, providing direct communication to pregnant women through SMS or interactive voice response (IVR), or on both the demand-side for clients and job aids for providers.

Five studies incorporated SMS messaging as a direct communication channel to pregnant women. In South Africa, LeFevre et al. [12] evaluated the Mobile Alliance for Maternal Action (MAMA) program, which delivered bi-weekly, gestational age-specific SMS messages to women registered at health facilities, covering danger signs, nutrition, and ANC reminders. In Iraq, Alhaidari et al. [28] implemented a facility-based intervention combining weekly SMS reminders for ANC visits with health advice, nutrition education, and a toll-free helpline, for $0.0225 per message. In Bangladesh, both studies by Jo et al. [10,25] evaluated the mCARE package, which included automated SMS reminders for clients alongside mobile-based pregnancy surveillance by community health workers. The 2019 study specifically compared two intensities: a comprehensive version (surveillance + SMS + home-visit reminders) versus a basic version with digital surveillance only. In Malawi, Larsen-Cooper et al. [11] assessed the Chipatala cha pa Foni (CCPF) program, a national toll-free maternal health hotline that delivered both voice and SMS tips and reminders to users.

Three studies from South Asia focused primarily on equipping community health workers with mobile applications. In India, two related studies evaluated the Reducing Maternal and Newborn Deaths (ReMiND) program. Prinja et al. [16] and Prinja et al. [29] examined a mobile app used by Accredited Social Health Activists for pregnancy tracking, ANC guidance, complication identification, and referral facilitation. Also in India, Saha et al. [26] assessed the Technology for Community Health Operations Plus (TeCHO+) mobile/web application, which enabled real-time data entry, high-risk case alerts, and supervision dashboards.

Two studies from West Africa evaluated comprehensive interventions that combined demand-side messaging with supply-side digital tools. In Ghana, Willcox et al. [27] assessed the Mobile Technology for Community Health (MOTECH) program, comprising "Mobile Midwife," an automated voice message service for pregnant women, and a Client Data Application for health workers that digitized records and generated care alerts. In Nigeria, Bowser et al. [13] evaluated a facility-based mHealth job aid using the CommCare platform, which supported health workers through task management and decision support for ANC.

Cost Components

The included studies varied in the scope of costs captured and analytical perspectives adopted, reflecting differences in intervention complexity and study objectives as shown in Table 2.

**Table 2:** Methodological characteristics of included studies ANC: antenatal care; LiST: Lives Saved Tool; CHW: community health worker; DALY: disability-adjusted life year; CEAs: cost-effectiveness analyses; CHRW: community health research worker; IVR: interactive voice response; SMS: short message service.

Study	Economic evaluation method	Perspective (with details)	Time horizon (duration costs/outcomes measured)	Discount rate (% applied to future costs/outcomes)	Costing approach	Model used	Sensitivity analysis parameters
Cost-effectiveness analyses (n=7)							
Saha et al. [26]	Cost-effectiveness analysis	Health system (program implementation + service delivery costs)	2 years	3%	Ingredient-based, bottom-up	Decision tree	Discount rate for costs and health outcomes; Useful life of capital items; Program costs; effectiveness estimates; Unit cost of services
Bowser et al. [13]	Cost-effectiveness analysis	Health system + societal (program costs + patient transport + lost wages)	1 year	None	Ingredient-based	LiST	Discount rate applied to costs; Useful life of equipment; Cost estimates of deliveries; Coverage levels of ANC interventions; Mortality rates
Prinja et al. [16]	Cost-effectiveness analysis	Health system + societal (program costs + household expenditures)	10 years	3%	Ingredient-based micro-costing	Decision tree	Discount rate applied to costs and health outcomes; Program costs; Service utilization rates; mortality reduction estimates; DALY weights and life expectancy values
LeFevre et al. [12]	Cost-effectiveness analysis	Societal (program + health system + user costs: transport, lost wages)	5 years	3%	Ingredient-based	LiST	Cost estimates, including program, health system costs, and user costs; Service utilization rates
Willcox et al. [27]	Cost-effectiveness analysis	Program (direct implementation costs only)	10 years	3%	Ingredient-based	LiST	Cost estimates, including personnel costs, and other program costs; Health effects, including children's lives saved, maternal lives saved, and the number of stillbirths averted
Jo et al. [25]	Cost-effectiveness analysis	Societal (program + provider + user costs)	4 years	3%	Ingredient-based	LiST	Cost estimates, including training, supervision, technology, mobile services, and CHW time; Service utilization rates
Jo et al. [10]	Cost-effectiveness analysis	Program (development, start-up, implementation costs)	10 years	3%	Ingredient-based	LiST	Cost estimates including training, supervision, technology, mobile services, CHRW time; Service utilization rates (ANC use, and facility delivery)
Cost analyses (n=3)							
Alhaidari et al. [28]	Costing study (feasibility)	Program + user (SMS costs + provider time not monetized)	1.5 years	None	Direct costing	None	Not done
Prinja et al. [29]	Costing study	Health system + patient (program + supervision + patient costs)	4 years	3%	Ingredient-based economic costing	None	Discount rate; Useful life of the software/program; Cost scenarios for software development; Key cost inputs; Hosting charges from the software provider.
Larsen-Cooper et al. [11]	Cost-outcome analysis	Program (total program costs allocated to maternal health)	22 months	3%	Program expenditure analysis	None	Call volume; Message success rate; Service modality (removing IVR and voice message components, leaving only the SMS text messaging); Mobilization and airtime costs; Cost scope
Summary (n=10)	7 CEAs, 3 costing studies	4 societal, 4 program, 1 health system	1–10 years	3% where applied	Varies by perspective; includes intervention delivery, personnel, equipment, and user costs (transport, time) where societal perspective is applied	5 of 7 CEAs used LiST	Varies - most commonly coverage indicators, intervention costs, and discount rates

Three perspectives were used across the studies. A societal perspective, capturing the broadest range of costs including patient-incurred expenses, was adopted in four cost-effectiveness studies [12,13,16,25]. Findings from the societal perspective show that, as demonstrated by LeFevre et al. [12] in South Africa, user costs (e.g., transport and lost wages) could account for up to 90% of total costs. A health system perspective, focusing on costs incurred by healthcare providers, was used by Saha et al. [26] and Prinja et al. [29]. A narrower program perspective, concentrating on direct implementation costs, was used in four studies [10,11,27,28].

All studies used an ingredient-based, bottom-up costing approach, though details varied. The cost categories captured included: start-up/development costs (e.g., software development, training, mobile devices); implementation/recurrent costs (e.g., personnel, supervision, SMS messaging, airtime, monitoring); service delivery costs (e.g., ANC drugs, lab tests, provider time), and user costs (e.g., transport, lost wages, household expenditures). The reported costs varied widely by intervention complexity and setting. At the lower end, Alhaidari et al. [28] in Iraq reported a cost of $0.0225 per SMS message, with a total program cost of $159.89. At the program level, Larsen-Cooper et al. [11] in Malawi reported a cost per user of $29.33, while Prinja et al. [29] in India estimated a cost per registered pregnant woman of $20.50, with scale-up costs projected at $2.77-$3.14 per woman. Time horizons ranged from short-term evaluations aligned with project cycles (one to two years) to extended horizons of up to 10 years designed to capture long-term mortality outcomes. For studies with horizons exceeding one year, a discount rate of 3% was uniformly applied to both costs and health outcomes, allowing for comparison across studies.

All studies except Alhaidari et al. [28] conducted sensitivity analyses to address uncertainty. Parameters varied included discount rates, useful life of capital assets, key program cost components, and, in cost-effectiveness studies, effectiveness parameters such as service coverage rates and mortality estimates.

Effectiveness Outcomes: ANC Uptake and Related Indicators

The included studies reported various effects of mHealth interventions on ANC outcomes, ranging from improvements in service coverage and quality to increases in knowledge and utilization at low to moderate cost. Details are presented in Table 3 below.

**Table 3:** Effectiveness and cost-effectiveness summary of included studies ANC: antenatal care; DALY: disability-adjusted life year; ICER: incremental cost-effectiveness ratio;  IFA: iron and folic acid; GDP: gross domestic product; GNI: gross national income; mCARE: mobile-based pregnancy surveillance and reminder system; SMS: short message service; UP: Uttar Pradesh; MNCH: maternal, newborn, and child health.

Study	ANC gain	ICER or unit cost	Other effects
Cost-effectiveness analyses (n=7)			
Saha et al. [26]	ANC coverage 80.1% vs 76.3%	$24/DALY; GDP per capita: $1,939	High-risk case detection: 22% vs. 2.67%
Bowser et al. [13]	ANC service coverage improved (tetanus toxoid, malaria prophylaxis, IFA); visit frequency not reported	$568/DALY (ANC only); $424/DALY (ANC + facility delivery); GDP per capita: $3,005	Cost per life saved: $13,739 (ANC only); $9,806 (ANC + facility delivery incentive)
Prinja et al. [16]	ANC exams +18.7%; IFA +12.7%	$205/DALY (health system); GDP per capita: $1,451.5	312 maternal deaths averted; 149,468 neonatal deaths averted; $425 million savings (societal perspective)
LeFevre et al. [12]	4+ ANC visits up 26% (from 46% to 72%)	$1,985/DALY (Year 1) to $200/DALY (Year 5): GNI per capita: $6,080	Immunizations: +5%; Societal cost at 60% coverage: $3.6M in Year 5
Willcox et al. [27]	No significant change in ANC visits detected	$21/DALY; GDP per capita: $1,480	59,906 lives saved over 10 years (6,298 maternal, 33,797 child, 19,811 stillbirths); Skilled birth attendance: +11%; facility delivery: +10%; measles immunization: +6%
Jo et al. [25]	ANC coverage projected 74% by 2027 (modelled)	$1,152/DALY (Year 1) to $462/DALY (Year 5): GDP per capita: $1,700	3,076 deaths averted (13× more than status quo)
Jo et al. [10]	Not reported	$31/DALY GDP per capita: $1,500	Comprehensive mCARE averted 354 more neonatal deaths than basic mCARE
Cost analyses (n=3)			
Alhaidari et al. [28]	ANC visits doubled (median 2 to 4)	Costing only: $0.0225/SMS	Total program cost: $159.89; Acceptability: >85% recommended program
Prinja et al. [29]	ANC visits increased (magnitude not reported)	Costing only: $20.50/pregnant woman	Cost per capita: $0.49/year; Scale-up cost (state-wide): $0.07–0.08 per capita; $2.77–3.14 per pregnant woman; Annual state-wide scale-up: $13.8–15.7 million (6% of UP's MNCH budget)
Larsen-Cooper et al. [11]	Knowledge of 4+ ANC visits up by 25% points; first trimester initiation +30% points	Costing only: $29.33/user	Total program cost: $287,357; Cost per successful contact: $4.33; Cost per additional user with improved outcome: $67–$355; 10 of 30 MNCH indicators improved significantly (15–80 percentage point gains); Cost per contact could drop 48% to $2.23 at full capacity

Several studies reported substantial increases in ANC service coverage. The MAMA SMS-based program in South Africa increased the proportion of women attending four or more ANC visits from 46% to 72% [12]. In Iraq, a simple SMS program doubled the median number of ANC visits from two to four per pregnancy [28]. The ReMiND intervention in India improved ANC indicators, including a 12.7% increase in iron-folic acid consumption and an 18.7% improvement in ANC examinations [16]. The mCARE intervention in Bangladesh was projected to achieve a 2.4-fold increase in ANC coverage over a decade [25]. The mHealth job aid evaluated by Bowser et al. [13] in Nigeria led to higher coverage of specific ANC services such as tetanus toxoid vaccination and syphilis testing.

Whereas the MOTECH program in Ghana did not report an increase in ANC attendance, the intervention still improved other key maternal health services, including skilled birth attendance (11%), facility delivery (10%), and measles immunization (6%) [27]. While Jo et al. [10] did not report ANC utilization data specifically, their comprehensive mCARE package was reported to have averted 354 neonatal deaths compared to the basic surveillance-only version.

Economic Evaluation Methods

The included studies used a range of economic evaluation methods, comprising seven cost-effectiveness analyses and three costing studies (two cost-outcome analyses and one economic costing study). A summary of the methodological characteristics is presented in Table 2.

The three costing studies focused on capturing implementation costs and intermediate outcomes. Prinja et al. [29] conducted an economic costing of the ReMiND program in India, and reported a cost per registered pregnant woman of $20.50 and a state-wide scale-up cost of just 6% of Uttar Pradesh's maternal and child health budget. Larsen-Cooper et al. [11] performed a cost-outcome analysis of Malawi's CCPF hotline and found a 25 percentage point increase in knowledge of recommended ANC visits for $29.33 per user, with potential for cost per contact to drop by 48% at full capacity. Alhaidari et al. [28] reported the leanest cost estimate, $0.0225 per SMS and $159.89 total program cost, alongside high acceptability (over 85% of participants recommended the program) and a doubling of median ANC visits from two to four.

The cost-effectiveness studies were based on diverse study designs, including a pre-post comparison [26], a randomized controlled trial [27], case-control designs [12,13], quasi-experimental designs [10,16], and one model-based analysis [25]. Two main modelling approaches were used to estimate health outcomes. The Lives Saved Tool (LiST) was used by five studies [10,12,16,25,27] to model maternal and child deaths averted and calculate disability-adjusted life years based on observed or projected changes in service coverage. Decision tree modeling was used by Saha et al. [26] and Bowser et al. [13].

All cost-effectiveness studies reported incremental cost-effectiveness ratios benchmarked against country-specific GDP per capita thresholds, following WHO-CHOICE guidelines [19]. The studies reported incremental cost-effectiveness ratios (ICERs) below the willingness-to-pay threshold of one times GDP per capita, and classified the interventions as highly cost-effective, although this threshold has been questioned in favour of context-specific or opportunity-cost approaches, especially in LMIC settings with constrained budgets [15,27]. Worth noting is the ReMiND program in India, which was found to be cost-effective from a health system perspective (ICER: $205/DALY averted) and cost-saving from a societal perspective, generating net economic benefits of $425 million by averting costly adverse outcomes [16]. Most evaluations compared the mHealth intervention against a paper-based system or standard government MCH program without an mHealth component. An exception was Jo et al. [10], who conducted a comparative analysis of two intervention intensities within the same platform.

Gaps Identified

The review identified several gaps in the existing literature. These have been summarized and presented in Table 4 below.

**Table 4:** Key research gaps identified across included studies ANC: antenatal care; CCPF: Chipatala cha pa Foni ("Health Center by Phone"); DALY: disability-adjusted life year; LiST: Lives Saved Tool; mCARE: mobile-based pregnancy surveillance and reminder system; NGO: non-governmental organisation; QALY: quality-adjusted life year; HIV: human immunodeficiency virus.

Study	Equity analysis	Long-term sustainability analysis	Study-specific gaps
Cost-effectiveness analyses (n=7)			
Saha et al. [26]	Not done	Not done	Relied on proxy data from a pilot project for software development costs instead of capturing actual expenditure; Inferred morbidity management costs from secondary literature rather than collecting primary data; Excluded disease complications from the decision model due to data unavailability; Used DALYs as the outcome measure because disorder-specific QALYs are not available in the Indian context; Calculated DALYs for low birth weight using a proxy condition (moderate motor impairment) rather than condition-specific disability weights; Did not conduct an equity analysis to examine how costs and benefits were distributed across socioeconomic groups
Bowser et al. [13]	Not done	Not done	Tracked only 5 of 10 major antenatal care interventions instead of capturing a complete picture of service delivery; Did not follow up on hypertension screening to confirm whether positive cases received appropriate management; Focused on ANC service delivery without integrating a demand-side component to effectively reduce unassisted home deliveries; Failed to examine whether benefits reached the poorest or most remote women, missing an opportunity for equity analysis; Relied on static costing assumptions instead of incorporating supply- and demand-side constraints into the model; Full compliance with ANC protocols assumed rather than verified
Prinja et al. [16]	Not done	Estimated scale-up costs under ideal conditions, but didn’t consider real-world conditions	Did not account for supply-side constraints that could limit the health system's ability to meet increased demand; Could not interview key officials involved in software design, potentially missing important cost inputs; Relied on retrospective cost data collection rather than prospective tracking of expenditures; Did not assess cost-effectiveness in low-burden settings, limiting generalizability beyond high-focus districts; Relied on modelled estimation of mortality reduction rather than empirical measurement of long-term outcomes
LeFevre et al. [12]	Not done (excluded HIV-positive subgroups despite high prevalence in the study population)	Not done (only 5-year forecast)	Relied on a retrospective case-control design with small sample sizes; Excluded a large proportion of interviewed participants due to incomplete health record data; Used modeled health effects in LiST based on utilization changes rather than measuring actual mortality reductionsWas not powered to detect statistically significant changes in individual immunization rates; Drew effectiveness estimates from less than 100 complete records per study arm rather than adequately powered samples; Based forecasts on observed enrollment patterns from a small pilot; Relied on patient recall for some services rather than verified clinical records
Willcox et al. [27]	Not done	Not done, only a 10-year projection of costs and coverage indicators	Cost data from a single district was used rather than representative data from diverse districts across Ghana; NGO personnel costs were used rather than actual government salary structures for projections; Researchers modeled a standalone system but should have evaluated integration with the government's existing system; costs modeled from a program perspective instead of societal or healthcare system; Health effects limited to outcomes with significant changes in trial; no measured impact on ANC visits
Jo et al. [25]	Not done	Not done, only modelled costs and health outcomes over 10 years	Years lived with disability were not included in DALY calculations due to lack of morbidity data; Linear interpolation of service coverage from baseline to target year assumed; Lives saved for children older than 1 year not included in LiST model
Jo et al. [10]	No equity analysis by geography or socioeconomic status	Not done, short-term pilot	A program perspective was taken, but household costs or service provision costs associated with the intervention were not included; Comparison group (basic mCARE) also used mobile phones for pregnancy surveillance, so benefits compared to paper-based status quo remain unknown; Statistical power was low due to small sample size; Direct benefits beyond mortality (improved communications, worker empowerment, enhanced accuracy, quality, and efficiency) were acknowledged but not quantified; Years lived with disability were not included in DALY calculations due to lack of morbidity data
Cost analyses (n=3)			
Alhaidari et al. [28]	Not done	Not done	Only reports marginal cost per message but omits critical inputs, e.g., setup, staffing, and overhead costs required to implement the program; Does not analyze the cost per additional antenatal visit achieved or the cost per adverse outcome averted, leaving the economic efficiency of the intervention unmeasured; Cost of text messaging intervention is not compared against any alternative strategies; Analysis does not quantify potential cost savings to the healthcare system from earlier problem detection or reduced emergency visits resulting from the intervention
Prinja et al. [29]	Not done	Not done, but modelled scale-up costs	The study relies on retrospective data collection; Detailed year-wise breakup of expenses during the implementation phase could not be obtained, requiring averaging of costs which may not reflect real annual variations; Lack of primary data on time spent by government officials on supervisory and monitoring activities, relying instead on assumptions from another study; Effectiveness values from the two pilot blocks may not be generalizable to the entire Uttar Pradesh state since these were specifically chosen from worst-performing high-priority districts; Analysis did not consider that technological advances over time may have reduced the prices of capital items such as mobile phones and software; Limited data available in Indian literature to assess the impact of mHealth interventions on out-of-pocket expenditures.
Larsen-Cooper et al. [11]	Not done	Not done, pilot costs only	Cost estimates likely exaggerated because total programmatic costs could not be disaggregated by service type; Analysis was unable to account for users who may have experienced more than one positive outcome as a result of using the CCPF service; Volunteer time was excluded from total costs because monetizing their contributions was not feasible due to challenges in separating their share of output or direct contributions; The cost-outcome analysis does not monetize the benefits of the program, which prevents comparison with the cost-effectiveness of other programs; The analysis only considers costs from the program provider perspective, omitting costs and cost savings from the perspective of users and the health system

A number of studies relied heavily on modelled estimates for long-term health outcomes rather than empirically observed data [12,16,25,27]. Several studies noted limitations related to retrospective data collection [12,29], small sample sizes with inadequate statistical power [10,12], and reliance on proxy data or assumptions where primary data were unavailable [13,26].

The reviewed studies are geographically concentrated, with studies from South Asia (India and Bangladesh) over-represented compared to sub-Saharan Africa, where maternal mortality burdens are highest. This limits generalizability across diverse health system contexts. A common gap across all studies was the lack of equity analyses, as none of the included studies disaggregated costs or outcomes by socioeconomic status, geography, or other vulnerability indicators.

Many studies assessed costs within donor funding periods rather than modeling long-term financial sustainability [13]. Scale-up costs were often estimated under ideal as opposed to real-world conditions [16]. The economic benefits appeared to strengthen over time, as reported for the MAMA SMS program, whose cost per DALY averted dropped substantially by Year 5 [12].

Methodological differences observed across studies, especially in the choice of perspective, time horizon, and modelling techniques, make it hard to compare costs and ICERs. In particular, the reported ICER range of $21 to $568 per DALY averted should be interpreted with caution, as these figures reflect different analytical perspectives, discount rates, and effectiveness assumptions and are not directly comparable across studies. They make it challenging to determine whether higher costs reflect greater program comprehensiveness or less efficient implementation.

Several studies acknowledged incomplete cost capture. Larsen-Cooper et al. [11] excluded volunteer time from total costs, Alhaidari et al. [28] did not monetize provider time, and Jo et al. [10] noted that direct benefits beyond mortality (e.g., improved communication, worker empowerment, enhanced efficiency) were not quantified.

Discussion

We reviewed evidence from seven cost-effectiveness and three costing studies. We mapped the scope and nature of evidence on the cost and cost-effectiveness of mHealth interventions aimed at promoting antenatal care uptake in low- and middle-income countries.

Summary of Findings

All cost-effectiveness studies reported ICERs below national thresholds of one times GDP per capita, making these interventions cost-effective or even cost-saving [12,16,25-27]. Most mHealth interventions were found to be associated with improvements in ANC-related outcomes, although the size of these effects varied by setting. Multiple studies reported substantial increases in service coverage, including higher rates of ANC registration, improved adherence to the recommended number of ANC visits, and greater consumption of essential supplements such as iron and folic acid [12,13,16]. The difference an intervention made seemed to depend on where it was implemented, and the biggest gains were seen in places with lower baseline coverage. In Iraq, where health system fragility may limit access, a simple SMS program doubled the median number of ANC visits at minimal cost [28]. On the other hand, in Ghana, where baseline ANC attendance was already high at 84%, no significant increase in attendance was reported on the MOTECH program, but the program was associated with improvements in other maternal health services, including skilled birth attendance and facility delivery [27]. This finding shows that mHealth interventions may work differently depending on existing health system capacity and coverage levels, as complements in high-coverage settings and as boosters in low-coverage settings.

Apart from ANC attendance, interventions that incorporated decision support for health workers demonstrated improvements in quality of care. The TeCHO+ and ReMiND programs in India facilitated early identification and management of high-risk conditions such as hypertension and anemia [16,26], while the mHealth job aid in Nigeria improved coverage of specific clinical services, including tetanus toxoid vaccination and syphilis testing [13]. These quality improvements are important as they contribute directly to averting maternal and neonatal morbidity and mortality, which are captured in DALY estimates.

Regarding economic value, the reviewed studies suggest that mHealth interventions fell below GDP per capita thresholds in all seven cost-effectiveness studies, though most of this evidence is derived from modelled rather than empirically observed outcomes, and should be interpreted accordingly. Costs ranged from affordable SMS programs at $0.0225 per message in Iraq [28] to more comprehensive platforms costing $20.50 per pregnant woman in India [29] and $29.33 per user in Malawi. This wide variation in costs is driven by differences in intervention intensity, scale, and analytical perspective. Studies adopting a program perspective, which focuses only on direct implementation costs, likely underestimate true resource use by leaving out health system and user costs, meaning that the lower end of the cost range should be interpreted with caution [11]. The reported ICERs, ranging from $21 to $568 per DALY averted across studies [10,16,25,26], were below the cost-effectiveness threshold of one times per capita GDP, classifying them as highly cost-effective according to WHO-CHOICE guidelines [19], though context-specific or opportunity-cost thresholds may be more appropriate in budget-constrained LMIC settings [13,15,26]. Even more interesting is the ReMiND program in India, which was found to be cost-effective from a health system perspective (ICER: $205/DALY averted) and cost-saving from a societal perspective, generating net economic benefits of $425 million by averting costly adverse outcomes [16].

The economic benefits appear to strengthen over time. The MAMA SMS program's cost per DALY averted dropped from $1,985 in Year one to $200 by Year five [12]. This means that the cost per outcome reduces as fixed start-up costs are spread over more users. Similarly, Larsen-Cooper et al. [11] projected that at full capacity, the cost per contact for Malawi's CCPF hotline could drop by 48% from $4.33 to $2.23. These studies show that while initial investments may be substantial, the long-term sustainability and value of these platforms improve with time and scale.

The available evidence shows that, whereas comprehensive interventions that combine things like SMS reminders, home visits, and worker training cost more upfront, they often deliver greater health gains in return. The mCARE studies in Bangladesh demonstrated that adding SMS and home-visit reminders to a basic digital surveillance system averted 354 additional neonatal deaths at an ICER of $31 per DALY averted [10]. Even so, the benefit resulting from the addition of extra components likely depends on baseline coverage and health system readiness. The success of these tools hinges not merely on the technology itself but on how well they are embedded within and enhance existing community-based health worker programs. Interventions that strengthened CHW capacity while also reaching women directly, such as MOTECH in Ghana [27] and mCARE in Bangladesh [10,25], appeared to achieve broader impacts beyond ANC alone.

We identified differences in approaches across studies, especially in the choice of perspective, time horizon, and modelling techniques. While this difference is expected, it makes comparison of costs and ICERs difficult across studies and settings. On the positive side, there was consistency in several important areas, for example, all studies used ingredient-based costing approaches, studies with horizons exceeding one year applied a 3% discount rate, and all cost-effectiveness studies benchmarked ICERs against GDP per capita thresholds. The common use of the Lives Saved Tool (LiST) across five studies [10,12,16,25,27] shows a possible emerging trend in modelling long-term health impacts. The heavy reliance on modelled rather than empirically observed long-term outcomes introduces uncertainty. While sensitivity analyses were applied to address this, primary data collection over extended time horizons would make findings more reliable.

We identified several gaps that future research needs to address. There is a lack of analysis focused on equity across all ten studies. None disaggregated outcomes or costs by socioeconomic status, geography, or other vulnerability indicators. As such, it remains unclear whether these mHealth interventions equitably improve ANC uptake across all population subgroups or risk worsening existing disparities by primarily benefiting more advantaged groups, those with better network access, higher literacy, or greater ability to act on information received. Healthcare needs to be inclusive; future studies explore how interventions affect specific sub-populations, in order to ensure that technology adoption is aligned with the human goal of leaving no one behind. Furthermore, the studies included in this review are geographically skewed, with studies from South Asia (India and Bangladesh) over-represented compared to sub-Saharan Africa, where maternal mortality burdens are highest. This limits generalizability across different health systems and contexts. The interventions that work in India's CHW-heavy system may not translate directly to settings with different community health architectures. Also, there is limited evidence on long-term sustainability. Many studies assessed costs within donor funding periods rather than modeling financial sustainability under government financing scenarios. It is important to note that whereas modeled projections demonstrated declining ICERs observed over longer time horizons, e.g., the MAMA and mCARE studies, none reported real-world evidence of sustained cost-effectiveness [12,13,16]. Scale-up costs were often estimated under ideal conditions without considering real-world conditions such as staff turnover, network reliability, or device maintenance [12,13,16]. Studies that modeled longer time horizons tended to show stronger economic benefits over time [11,12], implying that pilot or short-term evaluations may fail to fully capture the sustained impact and long-term value of these interventions. Finally, incomplete cost capture was acknowledged in several studies. Larsen-Cooper et al. [11] excluded volunteer time, Alhaidari et al. [28] did not monetize provider time, and Jo et al. [10] noted that direct benefits beyond mortality, such as improved communication, worker empowerment, and enhanced efficiency, were acknowledged but not quantified. Greater attention to established reporting frameworks, e.g., WHO-CHOICE [19], could improve completeness and comparability across future studies.

Comparison With Non-mHealth Interventions

It is hard to compare mHealth ANC interventions with non-mHealth alternatives due to the absence of a common comparator in the included studies. However, comparing the reported ICERs with the broader maternal health literature provides some useful insights. A systematic review of non-mHealth strategies to improve maternal and newborn health care in LMICs reported ICERs ranging from $3 to $302 per DALY averted for community-based demand and supply-side strategies targeting maternal and newborn health utilization and provision [30]. A recent scoping review of CHW-only reproductive, maternal, and newborn health programs in LMICs reported cost per DALY averted ranging from $64 to $478 [31]. The mHealth ICERs reported in this review ($21-$568 per DALY averted) are comparable to non-mHealth community-based strategies, suggesting that mHealth-enhanced ANC interventions can achieve value for money similar to traditional community-based approaches.

Limitations

This scoping review is not without limitations. By restricting the scope to English-language publications, we may have excluded relevant studies from non-English-speaking regions, introducing potential language bias. Besides that, the considerable difference in methods across included studies limits our ability to draw pooled conclusions about specific cost estimates or effect sizes. Furthermore, we did not assess the quality or risk of bias of individual studies. Five of the seven cost-effectiveness studies used the LiST, a modelling approach, rather than actual observed outcomes, which introduces uncertainty into the estimates. Also, the search was limited to three databases due to institutional access constraints, and while these are the most relevant for this topic, additional studies may exist in regional or discipline-specific databases. The geographic concentration of included studies in South Asia also limits the generalizability of findings across all LMIC contexts. Finally, the rapid pace of change in mobile technology means that interventions evaluated in earlier studies may not reflect current technological capabilities or costs.

Policy Implications

In spite of the identified limitations, the findings have clear implications for policymakers and program implementers. The consistent cost-effectiveness in different countries and intervention types provides economic justification for investing in mHealth as part of broader maternal health strategies. Investment decisions must be context-specific. In settings with low baseline ANC coverage, simple SMS reminder systems may offer rapid, low-cost gains. In settings with established CHW platforms, investments in mobile applications that improve worker capacity and data quality may yield broader improvements across the continuum of care. Investment in mHealth interventions needs to cover the full costs of implementation, including training, supervision, and periodic changes in technology, rather than focusing solely on initial development or pilot-phase costs. Investment decisions must consider not only effects but also whether benefits reach the most vulnerable women, especially those with limited mobile phone access, lower literacy, or living in hard-to-reach areas. Policymakers should therefore prioritize implementation research that accounts for differential impact across sub-populations, and consider additional strategies such as targeted interventions to ensure that even the most disadvantaged are reached.

## Conclusions

In reviewing the existing evidence on the cost and cost-effectiveness of mHealth interventions for ANC in LMICs, we found that these interventions are a cost-effective approach to improving ANC uptake, though this finding is largely based on modelled rather than empirically observed outcomes and should be interpreted cautiously. Across the seven cost-effectiveness analyses reviewed, reported ICERs consistently fell below national GDP per capita thresholds, with cost-saving potential from a societal perspective, presenting an economic justification for strategic investment in mHealth as part of broader maternal health strategies. The Lives Saved Tool was a common framework for modelling long-term health impacts across most studies, suggesting an emerging standard in assessing cost-effectiveness. However, several gaps remain, including equity analysis, long-term sustainability evidence, and the underrepresentation of sub-Saharan Africa. Cost-effectiveness analyses with a particular focus on equity are needed to ensure that future mHealth investments align with universal health coverage goals and benefit women of lower socioeconomic groups, for example, those with limited phone access, lower literacy, or in hard-to-reach areas. Future research should aim to standardize economic evaluation methodologies, so as to enable comparisons across studies, investigate potential differences in impact across socioeconomic groups, and generate longer-term evidence on sustainability and scale-up under real-world conditions. Addressing these gaps will be essential to ensure that the potential of mHealth translates into equitable and sustainable improvements in maternal health outcomes.
